# Environmental Risk Awareness Among Romanian High School Students Enrolled in Food-Related Study Programs

**DOI:** 10.3390/foods15112017

**Published:** 2026-06-04

**Authors:** Loredana Dumitrascu, Iulia Bleoanca, Daniela Borda

**Affiliations:** Faculty of Food Science and Engineering, Dunarea de Jos University of Galati, Domneasca Street 111, 800201 Galati, Romania; loredana.dumitrascu@ugal.ro (L.D.); iulia.bleoanca@ugal.ro (I.B.)

**Keywords:** TPB, SEM, low environmental awareness, high school students, sustainability

## Abstract

This paper evaluated low environmental risk awareness related to the food industry among students in Romanian high schools offering food-related educational programs, within the framework of Sustainable Development Goal 4 (SDG4). The study used a questionnaire based on 349 self-reported answers and interviews conducted with an independent group of 20 students. Using an informed theory of planned behavior (TPB) model and a structural equation modeling (SEM) approach, the influence of knowledge, attitude, and subjective norms on low environmental risk awareness was tested in order to understand the potential of Romanian high school students to become responsible food citizens. Subjective norms and knowledge showed significant effects on self-reported low environmental risk awareness (*p* < 0.001), indicating the need for interventions to consolidate pro-environmental social norms and offset low environmental risk awareness in Romanian high schools with food-related educational programs. The interviews supported the dominant importance of subjective norms over the other variables in relation to low environmental risk awareness and in shaping future food citizens.

## 1. Introduction

During the last 200 years, the food industry has played a crucial role in sustaining the evolution of urbanized humankind by providing valuable nutrients, convenience, and food safety to large populations. However, its fast-paced growth also led to evolutionary mismatch [1] and increased environmental burden, including greenhouse gas emissions, soil degradation, and excessive water losses [2]. The negative impact of the food industry on resources and its contribution to environmental degradation are often amplified by reckless consumer behavior [3]. While the passive consumer is driven by consumerism and embedded in and controlled by the industrialized food system [4], the food citizen, or citizen-consumer, is an individual who can drive changes in the food system through more sustainable food choices [5,6,7]. The younger generation is a key stakeholder, as it will carry the burden of past and present environmental negligence and, at the same time, act as a significant driver of behavioral change [8]. The responsibility of high school students, particularly those enrolled in food-related programs, is even greater, since they represent the food professionals of tomorrow and should be closely involved in taking active measures to support sustainability. Nonetheless, the complexity of food citizenship is shaped by a combination of knowledge, values, behaviors, and social contexts.

Intervention strategies for turning passive consumers into responsible food citizens are complex and intricate, mostly because eating is a highly habituated, cultural activity, and consumers find it difficult to change their food preferences and habits [9]. Policy interventions by public and private entities, together with early education, are crucial for inducing changes in consumers’ behavior [9,10]. Basically, the psychological and cognitive foundation for becoming future food citizens is related to an awareness of how food choices affect health, the environment, and society, as well as the ability to connect personal behavior with global outcomes.

In this context, educational interventions involve providing individuals with the knowledge, values, and skills needed to understand and evaluate the consequences of their food choices and future professional actions in promoting health, supporting societal development, and protecting the environment.

Several strategies have been adopted across the world to address sustainability issues. For example, Europeans recognize climate change as one of the most serious problems facing the world today and consider that education and training should prevail in providing learners and educators with knowledge and skills related to sustainability in order to respond to the planetary crisis [11]. UNESCO’s 2030 education agenda regarding the fourth Sustainable Development Goal (SDG4) also advocates that education for sustainable development is the key to unlocking progress and views it as a lifelong learning process and an intrinsic part of quality education that can simultaneously contribute to the achievement of all the other sixteen UN Sustainable Development Goals [12]. The educational system should act as a unit of knowledge and innovation, responsible for shaping the next generation of sustainability leaders while promoting initiatives for sustainable development [13]. Thus, educators bear the responsibility for adopting holistic approaches to facilitate understanding of the complexity of human systems by identifying accessible and inspiring communication strategies aimed at constructively addressing environmental issues [12]. The SDG4 educational framework provides guidance for ensuring that, by 2030, all learners acquire the knowledge and skills essential for a sustainable lifestyle. Especially for high school students enrolled in food-related study programs, this education will help them develop environmentally oriented abilities leading to sustainable societal change [14].

In Romania, the Ministry of Education acknowledged the need to prepare younger generations to respond to future challenges concerning climate change and implemented the educational program called Green Week in schools for the first time in 2021, aimed at reinforcing education for sustainable development. Between 2022 and 2023, a total of 863,910 activities were organized under the umbrella of this program. At present, the program, approved by Ministerial Decree no. 3.629/02.02.2023, consists of five educational days per year in each school for organizing field trips, workshops, debates, lectures, visits, or other activities. It also has a platform [15] with educational resources focused on raising awareness, educating students about climate change, and informing them about the Sustainable Development Goals. However, despite these interventions, research studies have shown that while some young individuals are more responsible and increase their civic engagement toward environmental sustainability, others react with disinterest and denial [8].

According to the latest data, in Romania, in 2025, the recyclability rate of food packaging increased to approximately 84% due to the implementation of programs such as RetuRo [16], but Romania still wastes around 2.5 million tons of food annually, equivalent to 150 kg per person [17], indicating that there is room for improvement in consumer practices regarding environmental protection. In addition, although the Generation Z cohort demonstrates more pro-environmental attitudes, unlike older individuals, they are more reluctant to promote behaviors that foster environmental sustainability [8,13].

Understanding high school students’ perspectives on pro-environmental behavior is of paramount importance as they are the future consumers and key drivers of societal changes in the food industry [18,19]. Existing studies on sustainability in Romania mainly focus on university students or young adults [20,21], while research examining sustainability awareness among high school students is scarce. This lack of data suggests a significant gap that, if left unaddressed, may increase vulnerability in shaping long-term pro-environmental behavior. Early-stage educational training plays a critical role in consolidating environmental risk awareness. To the best of our knowledge, no assessment has yet been conducted in Romania to evaluate the environmental awareness of high school students enrolled in food-related educational programs and their potential to become responsible food citizens shaped by knowledge, attitude, and subjective norms.

Thus, in order to bridge this gap, this study was designed based on a model informed by the theory of planned behavior (TPB), which allowed us to examine whether the interaction effects between knowledge, attitude, and subjective norms affect low environmental awareness among the younger generation.

## 2. TPB Informed Conceptual Model

Proposed by Ajzen [22], TPB is one of the best-known conceptual models applied in consumer studies, stating that the intention to undertake a certain behavior is influenced by personal attitude, subjective norms, and perceived behavioral control. The model proposed in this study is informed by the TPB [23] and is based on incorporating key cognitive and social determinants, such as attitude and subjective norms, which are core components in the formation of behavioral intentions. It was hypothesized that high school students who demonstrate stronger pro-environmental attitudes are more likely to exhibit pro-environmental behavioral intentions later on. Although the full TPB structure is not tested, this model focuses on earlier cognitive processes by examining how knowledge, attitude, and social norms contribute to counteracting low environmental risk awareness, which can be understood as an antecedent to intention and behavior within the TPB framework. Environmental awareness is an earlier cognitive stage that precedes stable intention formation, making it a more relevant outcome variable for younger populations. Moreover, high school students have limited autonomy over daily behaviors, less control over food-purchasing decisions, and no access to professional decisions. Thus, the use of a simplified TPB-informed model is more appropriate for high school students, who are still in a formative stage of cognitive and behavioral development. During high school education, behavioral intentions and perceived behavioral control are still developing, whereas knowledge accumulation, attitude formation, concerns, and sensitivity to social norms are more prominent. It is worth mentioning that this study focuses on low environmental risk awareness as an outcome, reflecting the early stages of belief formation that precede behavior. This approach is in line with previous research identifying knowledge, attitudes, and social norms as key determinants of behavioral control within the TPB model [23,24,25].

Knowledge was considered an additional construct in the model and the main precursor of behavioral change [26], as studies have demonstrated its importance in prioritizing actions [26,27,28,29,30]. This model is closely related to the models applied by Yadav and Pathak [18] and Chao and Zhang [31] to assess young consumers’ intention to buy green products.

### 2.1. Knowledge

Knowledge and awareness represent two distinct cognitive levels. Awareness can be described as a general understanding of a subject, whereas knowledge implies a deeper, more comprehensive, and detailed understanding [32]. The link between knowledge and pro-environmental awareness has been demonstrated by different studies [33]. This relationship is grounded in cognitive and environmental education theories, which underline that knowledge forms the basis for understanding environmental problems and risks. Thus, knowledge contributes to risk perception and awareness formation, although it may not directly translate into behavior. Knowledge is a foundational cognitive driver of awareness and may be considered an antecedent of behavioral intentions. Knowledge provides the informational basis for understanding environmental issues.

In fact, environmental concerns and knowledge often go hand in hand and sometimes influence people’s decisions to consume environmentally friendly products [34]. However, it has been well recognized that transmitting knowledge alone is not enough to change lifestyles and behavioral patterns [8,35,36,37], and that accurate information can sometimes be irrelevant for decision-making [38]. Studies have shown that increased environmental risk awareness may result from advanced environmental knowledge, but it is not sufficient to induce pro-environmental behavior [33,39] that should be reinforced by people’s beliefs, as these can modulate their intentions and behavior.

**Hypothesis** **1.** *A higher level of knowledge leads to greater environmental awareness and a lower tendency to disregard environmental risks.*

### 2.2. Attitude

Attitude is defined as the psychological tendency to favor or disfavor, to some extent, an entity, action, or decision [26,40]. This relationship between attitude and social awareness, viewed as a precursor of behavioral intentions, is consistent with the TPB. Attitude reflects inclination toward a certain behavior and involves evaluations based on beliefs, while a positive environmental attitude enhances sensitivity to environmental risks.

Thus, individuals with stronger pro-environmental attitudes toward sustainability are more likely to consider environmental issues important and are less likely to exhibit low environmental risk awareness or disregard environmental risks.

It was previously reported that even if a person knows what to do, she/he would not necessarily be inclined to do it [26,41,42]. Attitude is based on behavioral beliefs, meaning that teenagers are more likely to adopt environmentally sustainable behavior if it mainly produces positive outcomes, and their attitude toward these behaviors will be favorable [8]. Conversely, a negative attitude results from distrust and low environmental risk awareness, as also suggested by other researchers [43]. Students with a skeptical attitude question the magnitude of environmental risks and have low motivation to engage in pro-environmental behavior. High school students may perceive risks as distant and/or minimal when they have not directly confronted or fully understood the negative environmental effects. In this case, risks could be perceived as temporally, spatially, or socially distant.

Moreover, although the younger generation shows a more positive attitude toward environmental challenges, they often do not know the most effective actions for driving change and translate them into everyday actions [12].

Nevertheless, it has been reported that moral obligations and subjective norms also shape individuals’ environmental concern, thereby instilling a sense of responsibility for actions that contribute to environmental risk awareness [44,45].

**Hypothesis** **2.** *Stronger attitudinal distrust toward sustainability is associated with a higher tendency to display low environmental risk awareness.*

### 2.3. Subjective Norms

Subjective norms are defined as ‘perceived social pressure to perform or not perform the behavior [23] or as the degree of social pressure felt by individuals in the decision-making process. Subjective norms are the perceived opinions of significant others (family, friends, colleagues, related groups) that prompt the decision-maker to make corresponding behavioral decisions based on peer pressure.

High school students’ gregarious spirit depends heavily on their perceptions of social approval rather than on personal values, and in this context, subjective norms could either promote or inhibit environmental awareness [31]. During high school, individuals are particularly sensitive to social approval and group belonging, which means that perceived norms can strongly influence not only behavioral intentions but also earlier cognitive processes, such as environmental awareness.

The items included in this study are conceptualized as subjective norms rather than personal norms, as they refer to others’ perceived beliefs and opinions that could impact personal beliefs. Within our model, subjective norms describe the perceived social influence of relevant social groups and peers and do not evaluate students’ internalized moral obligations and sense of personal responsibility.

The items described as social norms focus on how respondents perceive their colleagues’ views regarding pollution in the food industry, thus indicating externally oriented social perceptions rather than internally held moral standards or obligations. Consequently, these items can be considered subjective norms, even if they reflect perceived peer beliefs shaped through the lens of the significant others rather than direct social pressure.

For high school students, in particular, peer perceptions strongly influence their cognitive evaluations, while the social context shapes how environmental problems are perceived. These social influences may play a significant role in increasing or decreasing environmental awareness among high school students.

Therefore, we could consider subjective norms as a social filter through which environmental risks are perceived. Moreover, subjective norms provide a conceptual and cultural foundation for students’ actions, which may stimulate their environmental awareness [46].

**Hypothesis** **3.** *Lower perceived environmental concerns across subjective norms are associated with lower environmental risk awareness (H3).*

### 2.4. Attitudinal Concerns on Sustainability and Environmental Knowledge

The relationship between attitude toward sustainability and environmental knowledge is well established in research [47,48]. Environmental knowledge is the basis for understanding environmental concerns, thereby shaping individuals’ beliefs. These beliefs influence the development of attitude, because individuals evaluate environmental risks and possible solutions. Nonetheless, attitude may also motivate knowledge uptake, as individuals with stronger pro-environmental awareness are more likely to seek information and engage with sustainability topics. This mutual relationship suggests that knowledge and attitude are interdependent constructs, especially among high school students.

Thus, the last hypothesis tested in this study was the correlation between attitudinal concerns toward sustainability and environmental knowledge, as these have been highlighted as two of the most significant dimensions of an individual’s environmental awareness [39]. Some studies indicate that while, for adults, positive attitudes toward the environment and sustainable development are more predominant than knowledge in predicting behavior, for high school students, attitude and knowledge have been reported as equal behavioral drivers [49,50]. Environmental knowledge and pro-environmental attitudes are interconnected, particularly for high school students who are searching for information about environmental sustainability issues [38].

**Hypothesis** **4.** *Attitudinal concerns on sustainability and environmental knowledge are correlated.*

### 2.5. Environmental Risk Awareness

Environmental risk awareness is considered a multidisciplinary concept situated at the crossroads of psychology, sociology, environmental science, and food science [31]. A low level of environmental risk awareness is associated with a limited ability to recognize the severity and likelihood of environmental risks, which may be reflected in people’s behavioral intentions related to environmental protection [31,51]. However, the terms environmental awareness and low environmental risk awareness, respectively, have no consensual definition and remain somewhat elusive, with interpretations varying in relation to individual ideologies [50].

The literature indicates that environmental risk awareness in general, or the perception of environmental risks associated with climate change, is evident in individuals with high levels of environmental knowledge, who are more likely to exhibit greater environmental concern, stronger behavioral intentions, and more sustainable behavior [52]. Our study evaluated the low environmental risk awareness as a dependent variable predicting behavioral intentions based on risk perceptions. Risk awareness is the perceived severity, likelihood, and personal relevance of environmental burdens. It does not include knowledge, attitudes, or social norms; rather, it reflects how individuals interpret and internalize environmental risk-related information.

The proposed model assumes that environmental knowledge, attitude, and subjective norms influence the formation of environmental risk awareness by shaping the interpretation of environmental concerns. A low environmental risk awareness could compromise behavioral intentions and hinder the development of young individuals into food citizens if not properly guided, thereby reducing their chances of becoming responsible food professionals in the future. Knowledge provides the informational basis for understanding environmental issues and assessing risks (H1), attitudes reflect evaluative judgments that shape the perception of risks (H2), and subjective norms introduce social influence that further frames individual interpretation (H3). In addition, knowledge and attitude are correlated (H4), reflecting the process of belief formation. Together, these constructs are conceptualized as antecedents that shape the development of environmental risk awareness by influencing how individuals process and interpret environmental information, which could shape behavioral intentions.

## 3. Research Methodology

This research applied a transdisciplinary approach, combining a quantitative online survey with qualitative face-to-face interviews.

### 3.1. Questionnaire Design

The items included in the questionnaire were based on several research papers addressing convergent topics, with a few additional changes made by the authors to adapt the items to the objectives of the current study. To document this process, a Supplementary file (Table S1) was created, indicating the key aspects derived from the literature [53,54,55,56,57,58,59,60,61,62,63,64]. Although the questionnaire initially had more questions, to ensure measurement adequacy, the final questionnaire included three items for low environmental risk awareness, four items for attitudinal concerns toward sustainability measures, five items for environmental knowledge, and three items for subjective norms (Table 1). Respondents rated each item on a five-point agreement scale ranging from 1 = strongly disagree to 5 = totally agree. The questionnaire was revised and approved by specialists with previous expertise in consumer behavior and survey-based research [65]. The specialists focused their evaluation on item relevance, wording clarity, and age-appropriate language. The study was approved by the Ethics Commission of the Faculty of Food Science and Engineering, “Dunărea de Jos” University of Galați, Romania (approval number: C2434). As the initial questionnaire was developed in English, it was first translated into Romanian by two specialists and then adapted to improve clarity and cultural appropriateness for Romanian high school students until consensus was reached.

### 3.2. Data Collection

Using a convenience sampling approach, an electronic questionnaire was distributed among high school students in grades 10 to 12 from high schools in Eastern Romania that have integrated food-related educational programs into their curriculum. The activity was conducted during the Green Week of the semester, when students are engaged in activities promoting sustainability, in this case, the sustainability of the food industry. These students are expected to become future operators, technicians, or even specialists in the food industry and are frequently informed about the environmental issues associated with it and taught concepts for promoting the sustainability of this sector. Moreover, modern teaching methods and school initiatives regarding environmental sustainability make the younger generation exhibit a higher propensity for environmental protection and greater awareness than previous generations [66]. The high school students were assisted by their teachers, who explained the procedure for filling in the questionnaire without interfering with decision-making and without commenting on the questionnaire content. The teachers’ role was to facilitate logistics and ensure a favorable classroom environment. When needed, researchers provided instructions and explained the aim of the study. The students were informed that there were no correct or incorrect answers in the survey and that there were no consequences associated with their participation. The questionnaire was anonymous and was completed individually, without discussion between students. Students provided informed consent, and the survey was accessed freely via Google Forms. A total of 380 answers were collected from 12 high schools in Galati, Focșani, Tecuci, Suceava, Fălticeni, and Rădăuți, all education institutions being situated in the Moldova region of Romania. After removing incomplete responses (20 with at least one item missing) and extreme outliers (11—based on Mahalanobis distance, for cases exceeding the critical χ^2^ value at *p* < 0.001, df = 15), a total of 349 responses were considered for the analysis prior to hypothesis testing. Although the use of a convenience sampling approach may restrict the generalizability of the findings, several authors [18,67] have suggested that using student samples can generate reliable research findings.

### 3.3. Qualitative Research

Qualitative research was performed in order to support the findings of the questionnaire and to enable an understanding of how knowledge, attitude, and subjective norms could influence students’ perceptions of environmental awareness [26].

#### Interviews to Evaluate Environmental Awareness Among High School Students

Two researchers specializing in consumer science and food-related environmental aspects from the Faculty of Food Science and Engineering, Dunarea de Jos University of Galati, Romania [65] conducted semi-structured interviews with an independent group of 20 twelfth-grade students (15 girls, 5 boys). During the interviews, no leading questions or preconceived hypotheses were formulated, and students were neither contradicted nor judged during the discussions. The semi-structured interview process, based on open-ended questions, lasted half an hour. All participants were informed prior to the interview about the research objectives, methodology, anonymization procedures, and the fact that they could withdraw from the interview at any time. All interviewed high school students signed an informed consent form stating their agreement to take part in the interview and their consent to data processing. Participants’ names were recoded to ensure the confidentiality of the interviewees. The qualitative component of the interviews was designed to explore students’ perceptions of environmental risks, peer influence, and family practices, and the role of school initiatives in understanding environmental risks related to the food industry. The interview protocol included open-ended questions such as: “Can you describe how often you recycle food packaging after consumption and explain the reasons behind your behavior?”; “What environmental practices are encouraged at home?”; “How do your friends view food waste, recycling, and composting?”; and “What initiatives are implemented in your school to promote sustainability?”.

Control questions were also included to check credibility, for example: “Can you provide an example of the last activity or lesson about sustainability that you remember clearly?” and “Please give a detailed example of the most recent occasion when you recycled an item. What did you do?” The interviews were conducted face-to-face. Subsequently, data analysis followed an inductive approach. The answers were audio recorded; however, due to their sensitive nature, the raw interview transcripts are not publicly available.

### 3.4. Data Analysis

First, a reliability analysis based on Cronbach’s alpha coefficient was conducted to measure the internal consistency of the measurement model, with a threshold value of 0.7 considered acceptable. Descriptive statistics, in terms of the mean and standard error of the mean, were calculated for all investigated variables (Table 1). Exploratory factor analysis (EFA) was applied to evaluate the underlying structure among the variables, using maximum likelihood and varimax as the extraction and rotation methods, respectively [24]. Factor extraction was determined by considering eigenvalues above 1. Items were retained based on communalities above 0.3. Convergent validity was established through factor loadings above 0.5, while discriminant validity was assessed using the pattern matrix. The appropriateness of the data for factor analysis was measured based on the Kaiser-Meyer-Olkin measure of sampling adequacy (KMO) and Bartlett’s test of sphericity. Descriptive statistics and EFA were conducted using SPSS Statistics 20 (IBM Software Group, Chicago, IL, USA).

Confirmatory factor analysis (CFA) was employed to assess the validity of the latent structure resulting from EFA, allowing the identification of variables expected to load onto a specific latent construct [68]. Convergent validity was evaluated using the average variance extracted (AVE) and composite reliability (CR), while discriminant validity was examined through the comparison of AVE and maximum squared variance (MSV). Structural equation modeling (SEM) examined the relationship between attitude toward sustainability measures, environmental knowledge, social norms, and environmental risk awareness. Model fit was considered acceptable based on the threshold values of the following parameters: Chi-square to degrees of freedom ratio (χ^2^/df) < 5, comparative fit index (CFI) > 0.8, Tucker–Lewis Index (TLI), and root mean square error of approximation (RMSEA) < 0.1 [26]. CFA and SEM were performed using AMOS 20 (IBM Software Group, Chicago, IL, USA).

## 4. Results and Discussion

### 4.1. Descriptive Statistics

The demographic profile of the respondents indicates balanced gender representation, with 55.6% female and 40.7% male respondents, while 3.7% of respondents preferred not to declare their belonging to an identification group. The age distribution of the participating high school students was uniform: 16 years old (27.8%), 17 years old (34.67%), and 18 years old (37.54%).

### 4.2. EFA

EFA was employed to identify the underlying factor structure of the data set. The component loadings, KMO, Bartlett test of sphericity, along with Cronbach’s alpha coefficient, are presented in Table 2. The KMO yielded a value of 0.811, suggesting meritorious sampling adequacy, while Bartlett’s test of sphericity was significant (*p* < 0.001), confirming the suitability of the data for factor analysis. The four factors explained about 57% of the total variance, which is higher than the minimum threshold value of 50% [69]. All items showed loadings above 0.5 on their factors, suggesting good convergent validity. Discriminant validity was confirmed, as no cross-loadings were identified in EFA, with the items being strongly related only to one extracted component.

### 4.3. CFA

**Figure 1:**
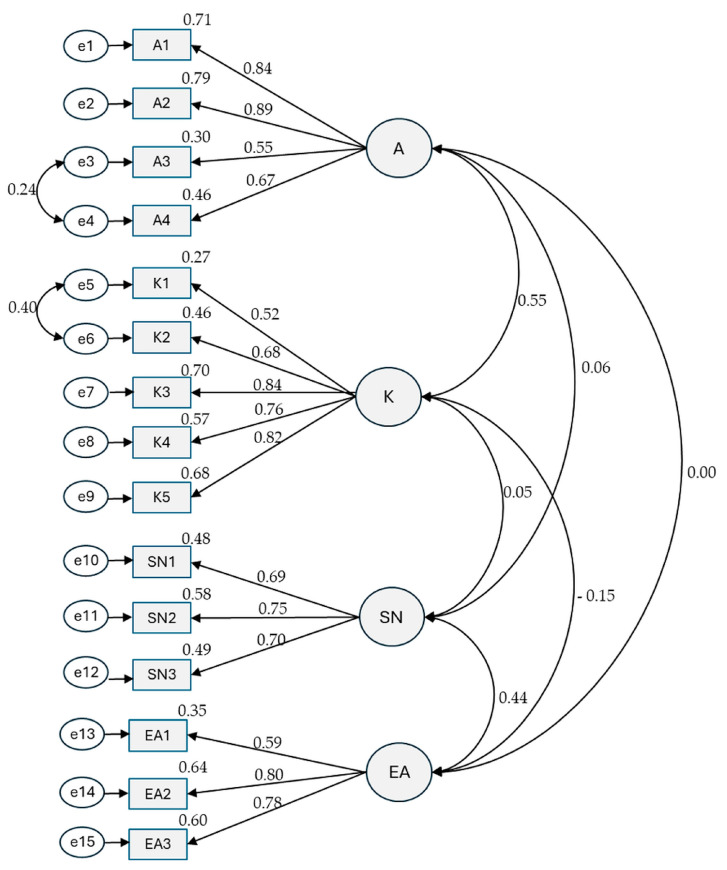
CFA model.

CFA (Figure 1) was employed to identify the correlations between the tested variables and to provide preliminary support for the factor structure extracted through EFA [24]. The final measurement model achieved an adequate fit (χ^2^/df = 2.42), as indicated by the non-normed fit indices (CFI = 0.95, TLI = 0.931) and by the RMSEA value of 0.06.

The model validity and reliability coefficients are presented in Table 3. CR was above 0.7, AVE was higher than 0.5, and MSV was lower than AVE, confirming reliability and validity. Since some of the items had similar meanings, a slight adjustment to the model fit was needed; therefore, the error variances for two pairs of items were correlated. For attitude, the correlation was made between items A3 and A4, as both highlight the feasibility and perceived effectiveness of the SDGs. For environmental knowledge, a correlation was made between K1 and K2, as both items capture content regarding greenhouse gas mitigation, reflecting shared variance associated with knowledge of emission-reduction targets and mechanisms. A sensitivity analysis comparing CFA models with and without correlated residuals indicated negligible differences in fit indices.

### 4.4. SEM

**Figure 2:**
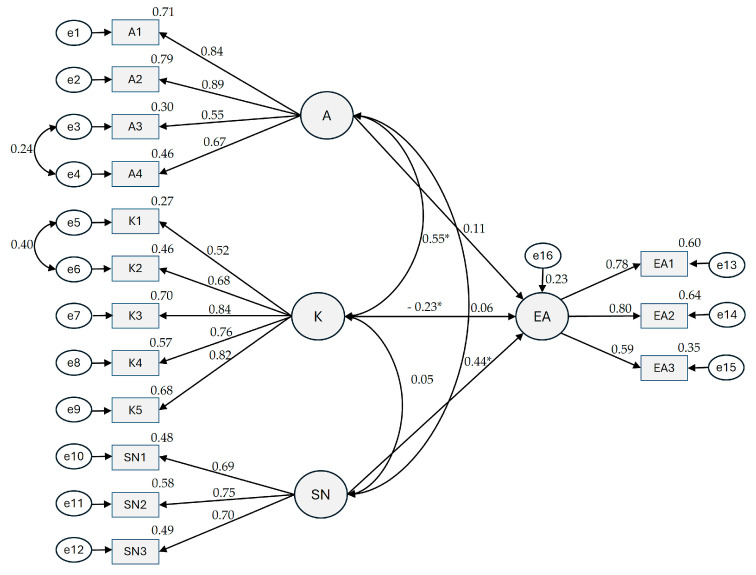
Structural equation model showing the relationships between attitudinal concerns toward sustainability measures, environmental knowledge, subjective norms regarding pollution minimization, and low environmental risk awareness * (*p* < 0.001).

Hypothesis 1, suggesting that environmental knowledge influences low environmental risk awareness, was accepted (β = −0.23; *p* < 0.01) (Figure 2). The negative effect indicates that high school students with increased environmental knowledge are aware of the risks associated with inaction and are less likely to support self-remediation theories beyond the resilience threshold of ecosystems, calling for immediate action in Romania. On the other hand, the optimistic bias of high school students enrolled in food-related educational programs in Romania may stem from a lack of knowledge or from overlooking or minimizing long-term risks when judging the environmental burden of the food industry. Thus, these students are less likely to become responsible food citizens if they do not have enough information about the need to limit the negative consequences associated with environmental damage caused by the food industry. Then again, students with increased knowledge could turn into food sustainability leaders by rationally weighing the environmental burden, thereby contributing to a more responsible and better food system in the future. Students’ environmental knowledge provides a basis for action strategies that shape intentions and attitudes through underlying belief systems, contributing to raising environmental risk awareness [67]. Conversely, Afroz et al. [70] found a negative effect of knowledge on sustainability and practice levels among students and highlighted the need to translate knowledge and attitude regarding sustainable development goals into actions that will raise environmental awareness and change students’ behavior and practices.

Hypothesis 2, stating that attitudinal distrust toward sustainability measures may have an effect on low environmental risk awareness, was not supported by our model (β = 0.11; *p* > 0.05). High school students’ intentions are not easy to predict, as their attitudes are not always reflected in their actual intentions [71]. High school students’ attitudinal concerns toward sustainability measures could probably be better cultivated by teachers by highlighting good practices, together with exercises designed to improve the ability to visualize the future, thus leading to a reduction in the underestimation of environmental risks. Researchers have also underlined that changing youths’ practices is a form of cultural shift, which is much more challenging than raising environmental awareness [72,73]. It is worth mentioning that authors have often pointed out the ambiguity in how sustainability might be perceived and understood among students [74], and in this case, only food-related sustainability measures were discussed.

The subjective norms associated with minimizing the effect of pollution were found to have the strongest positive significant impact on low environmental risk awareness (β = 0.44; *p* < 0.001), thus supporting Hypothesis 3 and indicating that the perception of subjective norms, or the social pressure to perform or not perform an action, influences low environmental risk awareness [74]. Social science research suggests that shifting social norms is a promising approach for generating the behavioral change required to achieve sustainability [75], but at the same time, negative pressure from social norms could translate into disregard for environmental protection measures and low environmental risk awareness. This suggests that in Romanian high schools with food-related educational programs, a social climate characterized by skepticism and a lack of support for measures aimed at reducing pollution associated with the food industry is present and negatively influences students’ environmental risk awareness and education. Unfortunately, this influence proved even stronger than the knowledge provided, and without the support of social norms, knowledge itself could become ineffective. Moreover, the positive estimate indicates that social norms that minimize the impact of pollution feed the idea that low environmental risk awareness should not be considered a priority, because these problems would resolve naturally without intervention. Similar to our study, Chao and Zhang [31] showed that subjective norms had the highest significant impact on environmental awareness, while Plohl et al. [76] highlighted that the people surrounding individuals are an important component in predicting environmental behavior. Social pressure is a significant predictor in explaining low environmental risk awareness among Romanian high school students, and, what is more worrisome, attitudes and habits, alongside values formed during youth, determine people’s behavior in later adulthood [75]. This could have serious long-term effects if we consider that high school students are generally highly susceptible to the influence of social norms.

High school students are motivated to follow the rules and standards set by the group to which they belong in order to remain part of that group [77]. Farrow et al. [78] highlighted that the effect of social norms should also take cultural factors into account, as differences can vary from one country to another, as indicated by Soon [74].

The model (Figure 2) displays a significantly positive correlation between attitudinal concerns regarding sustainability measures and environmental knowledge (r = 0.55; *p* < 0.001) among participants, thereby supporting Hypothesis 4. This suggests that when high school students’ knowledge of environment-related aspects improves, they tend to adopt an attitude that promotes sustainability measures. For example, a high school student with greater environmental knowledge can adopt a positive attitude regarding the recycling of used packaging materials. A high school student aware of the negative effects of greenhouse gases could more knowingly support actions aimed at reducing their impact on the environment. Knowledge regarding ways to reduce pollution can be translated into a positive pro-environmental attitude. The importance of environmental knowledge in shaping attitude toward sustainability was recently reported by Jillani et al. [66]. Pothitou et al. [33] linked the level of knowledge on greenhouse gas emissions with a more positive attitude toward reducing energy consumption, whereas Ajzen et al. [36] found that environmental knowledge correlated significantly with attitude toward sustainability measures.

Educational institutions are key providers in equipping students with knowledge that will engage them in a more pro-sustainable attitude. This goal can be achieved by applying innovative, effective teaching methods such as project/simulation-based learning, service learning, or gamified learning. In addition, informal learning by engaging in extracurricular initiatives, study visits, and volunteering actions could enhance knowledge acquisition while developing skills that will change students’ attitudes and behaviors related to environmental sustainability [13].

### 4.5. Predicting the Environmental Awareness of Romanian High School Students

The resulting SEM model showed a squared multiple correlations value (R^2^) of 0.23 (Figure 2). This suggests that environmental knowledge and subjective norms explained 23% of the variance in environmental risk awareness among Romanian high school students enrolled in food-related educational programs. Although in behavioral standards, a value of R^2^ > 0.2 is considered high [26], in our model, environmental knowledge and subjective norms on minimizing pollution were considered to have a moderate level of explanatory power. Addressing the urgent sustainability obstacles of the present can be achieved only by better understanding the concept of sustainability, and the key role is played by education [13]. Education for sustainable development is an essential component in fostering long-lasting and transferable knowledge promoting sustainability among students, with educators acting as catalysts of change [12]. Similarly, Leiva-Brondo et al. [79] highlighted the need for training and awareness-raising activities to improve sustainability education strategies in schools. Compared to knowledge, subjective norms were the strongest predictor of low environmental risk awareness, indicating that in-group sources are considered more trusted and influential than knowledge itself [80], and this was also the case among Romanian high school students enrolled in food-related study programs. Similar results were reported by Tamar et al. [81], where knowledge had the smallest effect on environmental behavior compared to subjective norms. Chao and Zhang [31] reported that subjective norms can influence the performance of environmental awareness, as consumers’ actions are more influenced by perceptions of social approval rather than by their personal values. Our findings show that, during the high school developmental stage, subjective normative influence tends to outweigh cognitive factors such as knowledge. Students enrolled in food-related programs in Romania demonstrated age-specific sensitivity to social identity dimensions that largely contribute to pro-environmental behavior. The dominance of subjective norms may also indicate a weaker internalization of environmental values, and this could affect the stability of pro-environmental behaviors over time.

Therefore, in terms of directing environmental risk awareness in a positive direction among high school students, an effective strategy should highlight environmentally oriented intentions demonstrated by individuals with whom others can easily identify [82]. In addition, external factors such as family habits, environmental programs at school, or pro-environmental messages on social media could also reinforce the awareness of environmental risks.

However, knowledge of environmental risks remains also very important. According to Ojala et al. [43], if teachers want to promote hope concerning climate change, it is wise to consider the negative emotions provoked by information about these environmental risks and use them as “teachable moments”. In other studies, opinion leaders have been shown to generate environmental concern in university students, who feel encouraged to promote pro-environmental activities [71]. Moreover, it is essential to offer solutions and communication tools tailored to younger generations in order to effectively promote change and strengthen environmental awareness. This can be achieved by engaging not only consumers but also key stakeholders from education, industry, policymaking, and civil society, each of whom has different motivations, resources, and capacities for applying strategies that are effective enough to generate broader societal impact [9,39]. Other researchers have found that social norms-based interventions can have mixed effects if they are not designed for a specific age group and have confirmed that social norms are likely to change over the course of adolescence [75], while our findings suggest that interventions and pro-environmental social norms are critically needed in Romanian high schools with food-related educational programs if we want to shape the food citizens of tomorrow.

As noted by other researchers, the main challenge in educating high school students is to increase their levels of environmental knowledge [25]. The relationship among knowledge, attitude, and subjective norms is complex; thus, the integration of sustainable development into education must consider and address that complexity, including greater engagement of educators in programs promoting the SDGs, particularly in food-related study programs.

The application of a sustainable development intervention model integrating psychological theories and teaching-learning strategies was recently indicated by Baena Morales et al. [12] as a flexible reference model that may promote environmental risk awareness and can be adapted to different educational programs. This model incorporates the development of competencies fostering critical, reflective, and systemic thinking and values, while aligning them with students’ sustainable development needs. In the Romanian context, this indicates that interventions should integrate peer-led and school-oriented approaches, such as the Green Schools platform. This initiative supports teachers in applying innovative strategies dedicated to eco-education by engaging teenagers in solving environmental problems through experiential learning conducted both inside and outside the classroom [83]. Let’s do it Romania is another platform that organizes educational campaigns and recycling projects aimed at increasing environmental awareness and promoting a sustainable lifestyle by mobilizing communities of all ages across Romania [84]. In a country like Romania, where citizens are among the least informed in Europe regarding environmental issues and risks, the young generation displays some of the lowest levels of ecological engagement in Europe, while these types of initiatives bring a new vision for addressing environmental education that will reinforce shared norms [84].

Although environmental risk awareness can be considered an antecedent of behavioral intention contributing to consolidating the development of future food citizens, and although other studies have also indicated that high school students’ level of pro-environmental attitude can effectively predict their pro-environmental behaviors [24], further studies should confirm the mediating power of the environmental risk awareness variable in predicting the behavior of young adults in Romania. This requires the long-term engagement of educators and trainers to ensure the effectiveness of environmental education in food-related programs in Romania and to be able to deliver sustainability. The high school educational environment should valorize students’ primary interests and guide young people’s values by supporting their development toward becoming food citizens [59].

### 4.6. Observational Case-Studies with High School Students Enrolled in Food-Related Educational Programs

During the discussions, students collectively acknowledged the need for everyone to be involved in recycling and environmental protection in order to achieve the Sustainable Development Goals.

One male participant, Vlad (18 y.o., who grew up in an urban environment), declared he did not want to recycle polyethylene terephthalate (PETs) or other plastic-based containers due to idleness. The same attitude is favored by his close friends, while his family is not at all concerned with recycling. When he was asked, he replied that he “did not find recycling rewarding in any way”.

The school setting is a central socialization context in which young people develop their identity, academic, social, and emotional skills [85]. Most of the time, high school students tend to act in defiance of rules because they desire to run their own lives, make their own decisions, and want to be highly regarded by their peers while also being different from them [86]. According to Ragelienė [87], the main task of high school students is to solve the crisis of identity versus role confusion, while declines in social responsibility seem to go hand in hand with declines in ecological assets [88]. Thus, to build social responsibility, affective bonds should be cultivated, and a rewarding climate for a proactive attitude toward taking care of the environment should be encouraged.

Another participant, Edi (18 y.o., who grew up in a rural environment), from a small rural village in Suceava county, said he “believed that only rich people can afford to throw away empty PET water bottles without worrying about recycling or where they discard them”. He saw them doing so while driving their expensive cars; thus, he associated this behavior with high wealth status.

A distorted set of values can be very confusing for young high school students’ brains during the phase of identity formation, especially when their friends and peers engage in questionable behavior. Thus, there is a need for consistent examples of positive behaviors and support from close ones to reinforce the set of values related to environmental protection and sustainability. Therefore, food citizenship responsibility should be developed by using role models and teaching better sustainability education, while also encouraging critical thinking about moral choices and professional responsibility among Romanian high school students.

One female student interviewee, Diana (18 y.o., who grew up in an urban environment), said she “used to recycle in primary school and gymnasium because it was a classroom initiative, and the money made from recycling was used for students’ common extracurricular activities. Unfortunately, now that she is in high school, she is no longer recycling because there is currently no such initiative at school, and she does not want to be considered “*silly*” by her colleagues.

On the one hand, this demonstrates the importance of subjective norms in pro-environmental behavior, but it also reinforces the idea that high school students are likely to adopt environmentally sustainable behavior when it produces positive outcomes. The results they obtain motivate students to support measures that reduce pollution caused by the food industry through recycling, and they feel rewarded for their actions. In fact, all the discussions with students during the interviews support the conclusions of the self- reported questionnaire by directly or indirectly suggesting that the most important aspect for reducing low environmental risk awareness is related to the influence of social norms on environmental awareness.

### 4.7. Limitations

The study included mostly students from vocational food-industry colleges who filled in the survey during the Green Week, which may bias the results and limit the generalizability of the reported findings. Future studies should either include social desirability controls or alternative administration settings to minimize this source of bias. The results should be interpreted as context-specific and not representative of Romanian high school students overall. Another limitation is that the low environmental risk awareness measured was related to intention and not to actual behavior; therefore, future studies should include students’ actual environmental behavior in relation to subjective norms, attitude, and knowledge. The explanatory power of the model could be improved by including additional constructs with a known impact on environmental behavior. Yadav and Pathak [18] suggested separate constructs for subjective and objective knowledge among young people when measuring environmental behavior, while Chao and Zhang [31] showed that personal and subjective norms should be treated separately when evaluating their effect on environmental behavior mediated by environmental awareness. Xu et al. [89] found that injunctive and descriptive norms have an impact on pro-environment intentions. Although the questionnaire was reviewed by specialists in consumer behavior, future studies could benefit from the inclusion of experts in high school students’ development or environmental psychology, as well as from pilot testing. Another limitation is that the findings should be interpreted in light of Romania’s specific social and cultural environment, characterized by relatively low environmental awareness and increased social pressure resulting from lower incomes in comparison with other Western European countries [90]. EFA, CFA, and SEM were performed on the same sample, which could reduce the strength of construct validation due to the absence of split-sample or independent-sample confirmation. Although the measurement model was based on previously validated scales, the use of a single dataset may increase the risk of overfitting. Future research should include the replication of the model using cross-validation or independent samples.

## 5. Conclusions

This research used a TPB-informed model to examine the factors influencing low environmental risk awareness among Romanian high school students enrolled in food-related educational programs. Using an SEM model, predictors of low environmental awareness related to the food industry were assessed. The findings revealed that attitudes toward sustainability measures and environmental knowledge among high school students receiving formal food-related education were significantly correlated. Furthermore, environmental knowledge and subjective norms associated with pollution reduction were significant predictors of low environmental risk awareness. Subjective norms were found to be more important than knowledge in reducing low environmental risk awareness. This highlights the need for pro-environmental social norms and educational interventions that may enhance environmental risk awareness in Romanian high schools receiving formal food-related education and shape high school students into responsible food citizens of tomorrow.

## Figures and Tables

**Table 1:** Descriptive statistics of the tested variables.

Item	Construct/Item	Mean	SE
Factor 1. Attitudinal concerns toward sustainability measures			
A1	I believe that, in Romania, the Sustainable Development Goals are implemented with delay.	3.07	0.061
A2	I believe that it is necessary to accelerate the implementation of the Sustainable Development Goals.	3.28	0.062
A3	I believe that not all the Sustainable Development Goals will be achieved by 2030.	2.82	0.052
A4	I believe that the Sustainable Development Goals are good, even if their implementation is not successful in Romania.	3.11	0.061
Factor 2. Environmental knowledge			
K1	By 2030, Romania must reduce greenhouse gas emissions by 55%.	2.58	0.063
K2	Using biodegradable materials significantly reduces greenhouse gases.	2.91	0.059
K3	Recycling can help reduce polluting effects.	3.32	0.065
K4	Microplastics pose a serious threat to human health and the environment.	3.12	0.063
K5	By reducing food waste, natural resources can be saved.	3.20	0.066
Factor 3. Low environmental risk awareness			
EA1	The environment has the capacity to regenerate, and problems will resolve themselves.	2.55	0.055
EA2	The environmental situation is not as serious as it appears.	2.54	0.056
EA3	I do not think pollution can really influence our lives.	2.34	0.061
Factor 4. Subjective norms on minimizing pollution			
SN1	My colleagues believe that the pollution produced by the food industry is high, but no action should be taken.	2.66	0.045
SN2	My colleagues do not think the pollution produced by the food industry is high.	2.77	0.044
SN3	My colleagues disagree with the idea that the food industry contributes to pollution.	2.64	0.047

SE—standard error of the mean.

**Table 2:** EFA with factor loadings, variance explained, and reliability.

Construct/Item		Factor Loading	Variance %	Cronbach’s Alpha
Factor 1. Attitudinal concerns toward sustainability measures			25.84	0.841
A1.	I believe that, in Romania, the Sustainable Development Goals are implemented with delay.	0.776		
A2.	I believe that it is necessary to accelerate the implementation of the Sustainable Development Goals.	0.852		
A3.	I believe that not all the Sustainable Development Goals will be achieved by 2030.	0.601		
A4.	I believe that the Sustainable Development Goals are good, even if their implementation is not successful in Romania.	0.689		
Factor 2. Environmental knowledge			16.10	0.860
K1.	By 2030, Romania must reduce greenhouse gas emissions by 55%.	0.659		
K2.	Using biodegradable materials significantly reduces greenhouse gases.	0.746		
K3.	Recycling can help reduce polluting effects.	0.750		
K4.	Microplastics pose a serious threat to human health and the environment.	0.737		
K5.	By reducing food waste, natural resources can be saved.	0.732		
Factor 3. Low environmental risk awareness			8.80	0.763
EA1.	The environment has the capacity to regenerate, and problems will resolve themselves.	0.573		
EA2.	The environmental situation is not as serious as it appears.	0.723		
EA3.	I do not think pollution can really influence our lives.	0.790		
Factor 4. Subjective norms on minimizing pollution			6.20	0.760
SN1.	My colleagues believe that the pollution produced by the food industry is high, but no action should be taken.	0.661		
SN2.	My colleagues do not think the pollution produced by the food industry is high.	0.761		
SN3.	My colleagues disagree with the idea that the food industry contributes to pollution.	0.668		
Total variance explained (%) = 56.94; KMO = 0.811; Bartlett’s test of sphericity <0.001				

EFA—Exploratory Factor Analysis; KMO—Kaiser-Meyer-Olkin measure.

**Table 3:** Model validity and reliability.

	CR	AVE	MSV	MaxR(H)	SN	A	K	EA
SN	0.761	0.515	0.191	0.764	0.718			
A	0.835	0.566	0.306	0.883	0.057	0.752		
K	0.850	0.537	0.306	0.875	0.048	0.553	0.733	
EA	0.769	0.530	0.191	0.792	0.437	0.001	−0.154	0.728

SN—subjective norms on minimizing pollution; A—attitudinal concerns toward sustainability measures; K—environmental knowledge; EA—low environmental risk awareness; CR—composite reliability; AVE—average variance extracted; MSV—maximum shared variance; MaxR(H)—maximum reliability.

## Data Availability

The data presented in this study are available on request from the corresponding author due to the attempt to protect the privacy of participants who are all minors.

## Supplementary Materials

The following supporting information can be downloaded at: https://www.mdpi.com/article/10.3390/foods15112017/s1. Table S1: Documenting literature sources of the questionnaire items. References [53,54,55,56,57,58,59,60,61,62,63,64] are cited in the supplementary materials.
