# Supplementary material for: Environmental Risk Awareness Among Romanian High School Students Enrolled in Food-Related Study Programs
Source: Foods. 2026 Jun 4;15(11):2017. doi: 10.3390/foods15112017 (PMC13257023; doi:10.3390/foods15112017)
Supplement: Supplementary file 1 [file foods-15-02017-s001.zip › foods-4296530-supplementary.pdf]

Supplementary Table S1. Documenting literature sources of the questionnaire items

Item	Variable	Supporting literature
**Factor 1. Attitudes on sustainability measures**		
A1	I believe that in Romania the sustainable development goals are applied with delay.	[53]<br>underlined the urgency of policy acceleration is supported
A2	I believe that is necessary to accelerate the implementation of sustainable development goals.	[54]<br>demonstrated people can hold positive attitudes toward SDGs necessity
A3	I believe that not all the sustainable development goals will be reached until 2030.	[55]<br>showed countries are not on track to meet the SDGs by 2030
A4	I believe that the sustainable development goals are good even if their implementation will not succeed in Romania.	[56 -57]<br>-anticipated delays in monitoring G20 SDG implementation<br>-indicated limited progress on SDG implementation in several countries
**Factor 2. Environmental knowledge**		
K1	By 2030, Romania must reduce greenhouse gas emissions by 55%.	[58]<br>-presented feasibility and achievement of SDGs, including climate goals
K2	Using biodegradable materials significantly reduces greenhouse gases.	[53]<br>-underlined the relationship between responsible consumption & production perceptions
K3	Recycling can help reduce polluting effects.	[59]<br>-demonstrated importance of awareness on sustainability actions such as recycling

K4	Microplastics pose a serious threat to human health and the environment	[60]<br>-showed perception differences across SDGs including environmental threats
K5	By reducing food waste natural resources can be saved.	[53]<br>-presented the responsible consumption dimension
**Factor 3. Environmental risks awareness**		
EA1	The environment has the capacity to regenerate and problems will fix from themselves.	[61]<br>-described environmental concern and optimism
EA2	The environmental situation is not as serious as it appears.	[62]<br>-suggested the role of optimistic bias in assessing environmental issues
EA3	I don't think pollution can really influence our lives.	[62]<br>- described the environmental attitudes inventory
**Factor 4. Subjective norms on minimizing pollution**		
SN1	My colleagues believe that the pollution produced by the food industry is high, but no action should be taken.	[63]<br>-presented normative beliefs & environmental behavior describing the „no action needed" approach
SN2	My colleagues don't think the pollution produced by the food industry is great.	[64]<br>-described subjective norms as: „People around me doubt that environmental problems are serious"
SN3	My colleagues are against the idea that the food industry contributes to pollution.	[53, 64]<br>-Indicated that several colleagues could reject pollution responsibility

53. Foroudi, P.; Marvi, R.; Cuomo, M.T.; Bagozzi, R.P.; Dennis, C.; Jannelli, R. Consumer perceptions of sustainable development goals: Conceptualization, measurement and contingent effects. *Br. J. Manag.* **2023**, 34, 1157–1183. https://doi.org/10.1111/1467-8551.12637.
54. Guan, T.; Zhang, Q. Value orientations, personal norms, and public attitude toward SDGs. *Int. J. Environ. Res. Public Health* **2023**, 20, 4031. https://doi.org/10.3390/ijerph20054031.
55. Pradhan, P. A threefold approach to rescue the 2030 Agenda from failing. *Natl. Sci. Rev.* **2023**, 10, nwad015. https://doi.org/10.1093/nsr/nwad015.

56. Oltra-Badenes, R.; Guerola-Navarro, V.; Gil-Gomez, H.; Vicedo, P. Do sustainable development goals (SDGs) influence the sustainability performance of organizations? *Sustainability* **2023**, 15, 1382. https://doi.org/10.3390/su15021382
57. Elder, M.; Newman, E. Monitoring G20 countries' SDG implementation policies and budgets reported in their voluntary national reviews (VNRs). *Sustainability* **2023**, 15, 15733. https://doi.org/10.3390/su152215733
58. Wang, B.; Chen, T. What do the sustainable development goals reveal, and are they sufficient for sustainable development? *PLoS ONE* **2024**, 19, e0310089. https://doi.org/10.1371/journal.pone.0310089.
59. Yuan, X.; Yu, L.; Wu, H. Awareness of sustainable development goals among students from a Chinese senior high school. *Educ. Sci.* **2021**, 11, 458. https://doi.org/10.3390/educsci11090458.
60. Bautista-Puig, N.; Barreiro-Gen, M.; Statulevičiūtė, G.; Stančiauskas, V.; Dikmener, G.; Akylbekova, D.; Lozano, R. Unraveling public perceptions of the sustainable development goals for better policy implementation. *Sci. Total Environ.* **2024**, 912, 169114. https://doi.org/10.1016/j.scitotenv.2023.169114
61. Franzen, A.; Vogl, D. Two decades of measuring environmental attitudes: A comparative analysis of 33 countries. *Glob. Environ. Change* **2011**, 21, 1001–1008.
62. Milfont, T.L.; Duckitt, J. The environmental attitudes inventory: A valid and reliable measure to assess the structure of environmental attitudes. *J. Environ. Psychol.* **2010**, 30, 80–94. https://doi.org/10.1016/j.jenvp.2009.09.001.
63. Fielding, K.S.; McDonald, R.; Louis, W.R. Theory of planned behaviour, identity and intentions to engage in environmental activism. *J. Environ. Psychol.* **2008**, 28, 318–326.
64. Poortinga, W.; Spence, A.; Whitmarsh, L.; Capstick, S.; Pidgeon, N.F. Uncertain climate: An investigation into public scepticism about anthropogenic climate change. *Glob. Environ. Change 2011*, 21, 1015–1024.
